# Sexual harassment among employees and students at a large Swedish university: who are exposed, to what, by whom and where – a cross-sectional prevalence study

**DOI:** 10.1186/s12889-022-14502-0

**Published:** 2022-12-01

**Authors:** A. Agardh, G. Priebe, M. Emmelin, J. Palmieri, U. Andersson, P-O Östergren

**Affiliations:** 1 Division of Social Medicine and Global Health, Department of Clinical Sciences, Malmö, Lund University, Malmö, Sweden; 2 Department of Social and Psychological Studies, Karlstad University, Karlstad, Sweden; 3 Department of Law, Lund University, Lund, Sweden

**Keywords:** Sexual harassment, University employees, University students, Gender, Academy

## Abstract

**Background:**

Sexual harassment (SH) in the workplace is prevalent and associated with poor health. Universities are large workplaces with complex formal and informal power relations, which may influence the prevalence of SH. Although employees and students share the university context, few studies on SH have included both groups. The overall aim of the study was to investigate SH among employees and students at a large Swedish public university regarding types of harassment, prevalence in different groups, characteristics of the perpetrators, and the circumstances in which it occurs.

**Methods:**

A cross-sectional analysis was performed, based on a web-based survey with 120 items that was sent out to all staff, including PhD students (*N* = 8,238) and students (*N* = 30,244) in November 2019. The response rate was 33% for staff and 32% for students. Exposure to SH was defined as having experienced at least one of ten defined SH behaviors during their work or studies.

**Results:**

Among women, 24.5% of staff and 26.8% of students reported having been exposed to SH. The corresponding figures were 7.0% and 11.3% for male staff and students and 33.3% and 29.4% for non-binary individuals among staff and students. Unwelcome comments, suggestive looks or gestures, and ‘inadvertent’ brushing or touching were the three most common forms of reported harassment, both among staff and students. Attempted or completed rape had been experienced by 2.1% of female and 0.6% of male students. Male and female perpetrators were reported by about 80% and 15%, respectively, of exposed participants. Among staff most reported events occurred during the everyday operation of the university, while among students the majority of the events took place during social events linked to student life. When exposed to a perpetrator from the same group (staff or students), women reported more often being in a subordinate power position in relation to the perpetrator.

**Conclusions:**

The results indicate that sexual harassment is common in the university context, and interventions and case management routines of events should consider power relations between victim and perpetrator, as well as the various contexts within which sexual harassment takes place.

**Supplementary Information:**

The online version contains supplementary material available at 10.1186/s12889-022-14502-0.

## Introduction

In our contemporary society universities are workplaces for many and also a work-like environment for their students. It is reasonable to assume that exposure to important psychosocial workplace environment factors such as sexual harassment (SH) occurs in a context partially shared by university employees and students. Therefore, it is important to investigate this issue jointly in both groups, as has been done in this study.

Workplace SH was recently found to be associated with a two- to three-fold increased risk of suicide or suicide attempts in a large population-based study, which had followed participants for 13 years [1]. This finding is not surprising, given that studies of workplace SH performed over several decades have consistently found SH to be related to negative health outcomes, above all, mental health problems [2–6], but also to hypertension and poor sleep [7] and to long-term sickness absence [8].

A recent systematic review investigated SH in higher education, and for exposed students the results indicated consequences such as anxiety, increased alcohol use, post-traumatic stress disorder, physical pain, and impaired career opportunities [9].

SH has been convincingly linked not only to gender-based power relations, but also to other types of power structures [3] and most likely a combination of those. University structures are characterized by a mixture of formal relations (such as institutional hierarchy, professional rank, and teacher–student relations) and informal power relations (such as career success and informal networks), which are potentially interesting for understanding SH, not only among employees, but also among students who share the same context.

Based on figures from the US, Clancy et al. assert that *‘Academia should lead and inspire change in other organizations. Instead, we have the highest rate of SH after the military’* [10]. This could be viewed as an uncomfortable paradox, true also in other settings and requiring urgent action. Such actions should be evidence-based, but despite many studies there are still considerable knowledge gaps. Firstly, most studies have been performed only on students. We know from other studies that SH is much more prevalent among women compared to men [11, 12] and among younger adults compared to older adults [13], which makes students a highly relevant group to target. We have also learned from previous studies that on average, one out of four female students has been exposed to SH, according to the review cited above [9]. The estimated prevalence varies due to time frame and geographical region. In a large study among university students in Norway, 21.6% of women and 5.7% of men reported having been sexually harassed within the past year [14]. However, larger contemporary studies from Swedish universities are lacking.

Few studies thus focus on SH among university staff. According to a review covering 86,000 respondents from 55 probability samples in the US, an average of 58% of women employed in the academic sector affirmed having experienced SH behaviors; this figure was higher than for women in the government and private sectors [15]. In another review describing workplace harassment among staff in higher education, it was noted that more elevated levels of harassment were found among managerial, administrative and professional staff compared to faculty, a finding that was thought to be related to gender issues [16]. In one study targeting all staff at a Swedish university, it was found that nine percent of women and two percent of men had experienced SH [17]. Of all 1719 new recipients of an academic award in 2006–2009, 62% responded to a survey on SH, and in this sample of clinician-researchers, 30% of women and 4% of men reported having experienced SH [18]. SH against members of the medical faculty was alarmingly frequent according to one recent study with a response rate of 26%; 82.5% of women and 65.1% of men reported at least one SH incident from students, staff or faculty during the past year [19].

Studies investigating university staff and students at the same time and place are even more rare. A survey sent out to 1,065 members of an association of women surgeons, which also included students, revealed that the majority of the 334 respondents affirmed gender-based harassment, which also included SH [20]. Recently, a prospective online study of SH experiences was performed, with a participation rate of 18% of all approached students and staff at the same academic medical campus in Florida. Overall, 52% of medical students, 31% of residents/fellows, and 25% of faculty respondents had experienced SH during the year 2018 [21].

The first studies on workplace SH focused only on women as victims of SH [22]. While most studies confirm that women encounter SH more often than men [23], 61% of men and 62% of women reported SH experiences on campus in one study [24]. With regard to the charges concerning SH filed to the US Equal Employment Opportunity Commission in 2020, 16.8% concerned men [25]. Due to the societal power imbalance between women and men, it has been considered unlikely that men’s experiences of SH will exactly mirror those of women [22, 26]. For example, men have reported that they feel harassed by negative stereotyping of men, as well as for deviating from the male gender role [27]. However, a ‘traditional’ SH behavior such as offensive staring or leering was experienced by as many as 30% of male responders in a study following 522 workers from 1988 to 2004 [28].

Other reported risk factors for experiencing SH, apart from female gender alone, have been female gender in combination with a supervisor position [28], an assertive and independent personality [29], and a university degree or being part of the highest occupational groups [13]. In a review of SH among students in the US, being young, White, transgender, or part of a sexual minority were all associated with higher rates of SH, as opposed to being older, of color, not transgender, and heterosexual [23]. Finally, SH has been reported to be particularly common among students in medical education [30].

For each workplace SH event, there is at least one corresponding agent of the offense, a perpetrator. Studies from Denmark and the US have indicated that in medical care, care of the elderly, social work, and customer service work, patients and clients may be the ones inflicting SH, as well as co-workers [12, 19].

In general, according to one review, perpetrators in academic settings are more likely to be male, White, known to the victim, and peers [23]. However, in one of the included studies female graduate students were at higher risk of being harassed by university staff than by peers [31]), and in another, a large on-line study, 19% of all students had reported experiencing faculty/staff-perpetrated SH [32]. In the Norwegian study mentioned above, faculty staff had been perpetrators in 0.6–4.6% of all instances of SH [14]. An inequity of power is undoubtedly at play in these cases. Information regarding the position of office of the perpetrator seems essential for understanding the SH phenomenon in a university setting.

Finally, when collecting knowledge regarding SH in higher education in the hope of developing preventive measures, it may also be helpful to clarify in what context and in which location the SH events occur [33].

The overall aim of the study was to investigate experiences of SH among employees and students at a large Swedish public university regarding types of harassment, prevalence in different groups, characteristics of the perpetrators, and the circumstances in which it occurs.

## Methods

### Design and study population

This is a cross-sectional study, performed within the framework of the ‘Tellus’ project at Lund University, Sweden, initiated in 2018 [34]. All staff, PhD students, and students at the university were invited to participate in a survey. The surveys, one for staff/PhD students and another for students, were sent by e-mail in November 2019. The original survey items in English were translated by the research team in a process involving both native Swedish and native English speakers. The response rate was 33% for staff/PhD students and 32% for students. After exclusion of those with missing data on both sex and gender (see below), age (*N* = 9 and 46, staff/PhD students and students, respectively) and those who did not answer any of the 10 questions on experiences of sexual harassment (*N* = 4 and 74, staff/PhD students and students, respectively), the final study population consisted of 2,736 staff/PhD students and 9,667 students.

There were no striking differences between the target population and the Tellus participants. However, the following observations could be made (Table 1): Women were slightly over-represented among Tellus participants, both among staff/PhD students, constituting 49.9% of the target population and 56.7% of the Tellus participants, and among students, constituting 55.4% and 62.6%, respectively, of the target population and Tellus participants. Staff/PhD student participants tended to be somewhat older than non-participants, with persons 41 years or older constituting 64.6% of the participant population and 57.1% of the target population. The reverse tendency was evident among students, with students 30 years or younger constituting 90.7% of the Tellus student population and 84.2% of the target population. Professional group affiliations were remarkably similar, especially since the diversity of occupations among university staff may entail differing classification by the university administration and by self-report. Staff with permanent (versus temporary) contracts constituted 60.6% of the target population and 72.3% of the Tellus participants. Students paying a university fee comprised 4.6% of the Tellus student participants and 3.5% of those in the target population.

**Table 1:** Characteristics of study participants and target population of staff & PhD students and students at Lund University

	University staff & PhD students				Students			
	All in target population		Tellus participants^a^		All in target population		Tellus participants^a^	
	n	%	n	%	n	%	n	%
Gender								
Women	4114	49.9	1551	56.7	16,744	55.4	6055	62.6
Men	4124	50.1	1161	42.4	13,500	44.6	3544	36.7
Non-binary	-	-	24	0.9	-	-	68	0.7
Total	8238	100.0	2736	100.0	30,244	100.0	9667	100.0
Age – Staff/PhD students								
≤ 30	1300	15.8	335	12.2				
31–40	2236	27.1	634	23.2				
41–49	1924	23.4	772	28.2				
50–59	1768	21.5	687	25.1				
≥ 60	1010	12.3	308	11.3				
Age – Students								
18–25					20,235	66.9	7488	77.5
26–30					5225	17.3	1280	13.2
31–40					2790	9.2	562	5.8
≥ 41					1994	6.4	337	3.5
Professional group *(missing among Tellus participants* = *2)*								
Professor	859	10.4	286	10.5				
Senior lecturer	1040	12.6	385	14.1				
Lecturer, Teaching Assistant	344	4.2	106	3.9				
Postdoc, Associate Senior Lecturer	509	6.2	161	5.9				
Researcher, Associate Researcher	1130	13.7	208	7.6				
PhD student, Research Student	1446	17.6	398	14.5				
Administrative staff, Library Staff	2058	25.0	788	28.8				
Technical Staff	735	8.9	311	11.4				
Other	117	1.4	91	3.3				
Employment form *(missing among Tellus participants* = *50)*								
Permanent	4993	60.6	1943	72.3				
Temporary	3245	39.4	743	27.7				
Paying student								
Yes					1061	3.5	444	4.6
No or No answer					29,183	96.5	9223	95.4

^a^After exclusion of participants with missing data; see Methods

### Background variables

*Gender* was assessed by two questions in the survey: ‘What gender were you assigned at birth?’ (female/male) and ‘what is your current gender identity?’ (Female/male/I do not identify as male or female). We used the answers to the second question to categorize participants as woman, man, or non-binary gender. However, when the answer to this question was missing (*N* = 15 for staff/PhD students and 84 for students), the answer to the first one was applied. Those who had refrained from answering both questions were excluded from the analyses (*N* = 3 for staff/PhD students, and *N* = 69 for students).

*Age* was categorized into groups, separately for staff/PhD students and students. *Country of birth* was recorded as ‘Sweden’, ‘Nordic countries (outside Sweden)’, ‘Europe (outside Nordic countries)’, or ‘outside Europe’. *Professional group among staff* was specified according to nine types: “professor”, “senior lecturer”, “lecturer/teaching assistant”, “postdoc/associate senior lecturer”, “researcher/associate researcher”, “PhD student/research student”, “administrative staff/library staff”, “technical staff”, and “other”. For the purpose of analyses these were then aggregated into six categories, “professor”, “senior lecturer”, “lecturer and researcher”, “PhD student/research student”, “administrative and technical support staff”, and “others”, on the basis of professional rank. Thus, the category “lecturer and researcher” included lecturer/teaching assistant, postdoc/associate senior lecturer, and researcher/associate researcher, and administrative/library and technical staff were grouped together as one category.

Staff/PhD students also answered one question about whether their *employment was temporary or permanent* and one question about whether they were in a *managerial position* or not.

Students self-reported as ‘*international student’*, yes or no, and also answered one question about having paid a *tuition fee* or not (only applicable for students with non-EU or non-Switzerland passports).

### Definition of sexual harassment (SH)

The ‘Sexual Experience Questionnaire’, with its three concepts *gender harassment, unwanted sexual attention,* and *sexual coercion* is one of the most widely used measures of SH [35], either as one of its complete versions, or as a number of ad hoc chosen items. It has also inspired the development of other questionnaires, such as the Bergen Sexual Harassment Scale [5] and the questionnaire used in the Norwegian study mentioned above [14]. A similar survey instrument was recently used in a study targeting all Canadian universities with medical schools [36]. However, in these and in many other studies originating outside the US, items covering ‘gender harassment’ are lacking [37]. According to Swedish law, there is a distinction between ‘harassment’ in general and ‘sexual harassment’ [38]. A construct of SH that does not explicitly include all types of gender harassment, but instead focuses on harassment with a clear sexual content may thus be more consistent with the perceptions of the general public regarding SH – even if these perceptions also differ, by gender and by age [39, 40].

After a review of the relevant literature and after the many focus group discussions with members of the target population that preceded this step, the following prerequisites for the current survey were agreed upon: it should a) cover both ‘everyday’ SH and sexual assault, b) cover both ‘traditional’ forms of SH and ‘new’ forms, such as on-line harassment, c) clearly indicate that the behavior was ‘unwanted’, and d) be possible to use also for men and LGBTQ individuals.

The following text introduced the survey section about experiences of sexual harassment (SH)*. ‘We will now ask some questions about your experiences of sexual harassment and sexual violence. Sexual harassment is defined as conduct of a sexual nature that violates someone’s dignity. This can be, for example, through comments or words, groping or indiscreet looks. It can also include unwelcome compliments, invitations, or suggestive acts. Sexual violence is defined in this study as attempts to conduct, or the conduct of sexual acts in which the person did not participate voluntarily. Have you experienced any of the following situations during your employment/your time as a student at Lund University?’.*

For the choice of potentially offensive situations, we used a modified version of the questionnaire employed by Phillips and co-authors in their study of medical students [36]. Firstly, the two items regarding inappropriate behavior received from patients in the medical consultation were omitted. Thereafter, the term ‘inappropriate’ was replaced throughout by ‘unwelcome’. Two further minor changes resulted in the following wording of the list of 10 items: unwelcome suggestive looks or gestures; unwelcome soliciting or pressuring for ‘dates’; unwelcome ‘inadvertent’ brushing or touching; unwelcome bodily contact such as grabbing or fondling; unwelcome gifts; unwelcome comments; unwelcome contact by post or telephone; unwelcome contact online, for example social media or email; stalking; and ‘attempts to conduct or the conduct of oral, vaginal, or anal sex or other equivalent sexual activity in which you did not participate voluntarily’ (hereafter labelled ‘attempted or completed rape’).When we refer to all 10 items in total, the term SH is used, although also rape (attempted or completed) is included.

In each case, the answer alternatives were ‘yes, once’, ‘yes, several times’, and ‘no’, and if ‘yes’, whether the event or the events had occurred during the past 12 months, one to three years ago, or more than 3 years ago. Those having answered ‘yes’ to at least one of these 10 questions were classified as exposed to experiences of SH and all others as non-exposed.

Several time frame options could be chosen. In this study we used an addition of events from all three mentioned timeframes, labelled “ever experienced”, for each of the ten SH items as well as for being exposed to at least one such item.

### Perpetrators, and location and context of SH

Since several occurrences of SH could have taken place in the context of the university, participants were instructed to select all alternatives that applied when describing SH events. Gender and position (or role) of perpetrator/perpetrators were established, as well as relationship of power (‘dominant/upper’ or ‘dependent/lower’ or ‘other’) between perpetrator and respondent. Further, location and context of SH events were assessed (see Additional file 1 for details).

### Statistical methods

The analysis was based on descriptive statistics presented as numbers and frequencies and chi square tests for assessing group differences. When considering details regarding characteristics of the perpetrator/perpetrators and the locations where the event/events took place, the total number of persons having affirmed any SH, i.e. *n* = 469 for staff, and *n* = 2,044 for students, were used as denominators. All analyses were performed using the IBM SPSS package, version 25. Significance was accepted at *p* < 0.05.

## Results

Having experienced at least one of the ten sexual harassment behaviors at least once during one’s employment or time as a student at Lund University was thus defined as exposure to SH. As seen in Table 2, this was the case for 17.1% of staff/PhD students and 21.1% of students. Non-binary persons reported the highest frequencies, with among 30% exposed, both among staff/PhD students and students. Among female staff/PhD students and students, 24.5% and 26.8%, respectively, had been exposed, while this was true for 7.0% of male staff/PhD students and 11.3% of male students. The gender-based differences for both staff/PhD students and students were statistically significant with *p*-values < 0.001.

**Table 2:** Type of sexual harassment ever experienced^a^ by university staff & PhD students and students, by gender

University staff & PhD students *(n of missing)*	Women *N* = 1551		Men *N* = 1161		Non-binary *N* = 24		Total *N* = 2736	
	n	%	n	%	n	%	n	%
Any of the following, hereafter classified as exposure to SH *(0*^*b*^*)*	380	24.5	81	7.0	8	33.3	469	17.1
Unwelcome comments *(25)*	256	16.7	42	3.6	6	26.1	304	11.2
Unwelcome suggestive looks or gestures *(6)*	231	14.9	28	2.4	4	16.7	263	9.6
Unwelcome ‘inadvertent’ brushing or touching *(10)*	131	8.5	21	1.8	5	20.8	157	5.8
Unwelcome soliciting or pressuring for ‘dates’ *(19)*	106	6.9	14	1.2	1	4.2	121	4.5
Unwelcome bodily contact such as grabbing or fondling *(8)*	80	5.2	17	1.5	2	8.3	99	3.6
Unwelcome contact online, for example social media or email *(13)*	60	3.9	18	1.6	1	4.2	79	2.9
Unwelcome contact by post or telephone *(18)*	52	3.4	18	1.6	2	8.3	72	2.6
Unwelcome gifts *(14)*	38	2.5	8	0.7	0	0	46	1.7
Stalking *(21)*	28	1.8	9	0.8	0	0	*37*	*1.4*
Attempted or completed rape *(16)*	6	0.4	3	0.3	1	4.2	10	0.4
Students *(n of missing)*	Women *N* = 6055		Men *N* = 3544		Non-binary *N* = 68		Total *N* = 9667	
	n	%	n	%	n	%	n	%
Any of the following, hereafter classified as exposure to SH *(0*^*b*^*)*	1625	26.8	399	11.3	20	29.4	2044	21.1
Unwelcome suggestive looks or gestures *(15)*	1026	17.0	167	4.7	16	23.5	1209	12.5
Unwelcome comments *(28)*	896	14.8	136	3.8	14	20.9	1046	10.9
Unwelcome ‘inadvertent’ brushing or touching *(27)*	790	13.1	167	4.7	8	11.8	965	10.0
Unwelcome bodily contact such as grabbing or fondling *(13)*	665	11.0	191	5.4	6	8.8	862	8.9
Unwelcome soliciting or pressuring for ‘dates’ *(13)*	499	8.3	71	2.0	10	14.7	580	6.0
Unwelcome contact online, for example social media or email *(19)*	400	6.6	55	1.6	5	7.4	460	4.8
Unwelcome contact by post or telephone *(28)*	130	2.2	23	0.7	3	4.5	156	1.6
Attempted or completed rape *(18)*	125	2.1	20	0.6	4	5.9	149	1.5
Stalking *(26)*	104	1.7	23	0.7	3	4.4	130	1.3
Unwelcome gifts *(27)*	66	1.1	12	0.3	1	1.5	79	0.8

*‘Ever experienced’* Experienced at least once during one’s employment or time as a student at Lund University

^a^See Methods for detailed description of wording in the survey

^b^Participants who did not affirm or refute any of the ten SH questions were excluded; *n* = 4 for Staff/PhD students and *n* = 74 for students

The three most common behaviors reported among staff/PhD students were, in descending order, unwelcome comments, unwelcome suggestive looks or gestures, and unwelcome ‘inadvertent’ brushing or touching. The pattern was similar for women and men, but with much higher prevalence figures for women; 16.7%, 14.9%, and 8.5%, respectively, versus 3.6%, 2.4%, and 1.8% for men.

Among female students, the three most commonly reported behaviors were the same as among staff/PhD students; however, the order was slightly different such that the most frequently reported behavior was unwelcome suggestive looks or gestures, reported by 17.0%. The most frequently reported behavior among male students was instead unwelcome bodily contact such as grabbing or fondling (5.4%), while both unwelcome suggestive looks or gestures and unwelcome ‘inadvertent’ brushing or touching were affirmed by 4.7%. Unwelcome comments were the fourth most common behavior, affirmed by 3.8% of male students.

Among non-binary individuals, the same behaviors as above were reported; however, one discrepancy noted was that among students of non-binary identification, unwelcome soliciting for pressure or ‘dates’ was one of the three most commonly experienced behaviors (14.7%).

One hundred and twenty-five female students (2.1%) reported having experienced attempted or completed rape during their time as a student at Lund University. The corresponding figure for male students was 20 (0.6%).

Among 5.7% of staff/PhD students and 14.9% of students the reported SH events had taken place during the past 12 months (see Additional file 2 for details regarding SH time frame exposures).

Table 3 presents the prevalence of ‘any’ SH experienced among staff/PhD students, stratified by gender and by age groups, country of birth, professional group, employment form, and managerial position. The variation by age was larger among men (*p* = 0.01) than among women. The men in the oldest age group, ≥ 60 years, reported the highest prevalence, 12.2%, while for women the age group with the highest prevalence, 27.8%, was 41–49 years of age. Persons born in Sweden and persons born in countries outside Europe tended to report higher prevalence than both those born in other Nordic countries and those born in other European countries, although this was not statistically significant; this was true for both women and men. Both among women and men, professors and associate professors reported a relatively high prevalence compared to the other professional groups; however, the difference was statistically significant among women only. PhD students among women and ‘researchers’ among men reported the lowest prevalence, 18.4% and 3.3%, respectively. Women with permanent contracts reported higher prevalence figures than those with temporary contracts (*p* = 0.002). The same tendency was present for men with permanent contracts and for men and women with managerial position versus those without such a position; however, these results were not statistically significant.

**Table 3:** Prevalence of sexual harassment ever experienced^a^ by background factors. University staff & PhD students; *N* = 2736

	Women				Men				Non-binary			All			
	Tot	Yes		P**	Tot	Yes		*p*	Tot	Yes		Tot	Yes		*P*
Background factors *(n of missing)*	n	n	%		n	n	%					n	n	%	
Age															
≤ 30	188	37	19.7		144	8	5.6		3	1	33.3	335	46	13.7	
31–40	373	93	24.9		250	10	4.0		11	3	27.3	634	106	16.7	
41–49	467	130	27.8		300	27	9.0		5	3	40.0	772	159	20.6	
50–59	365	82	22.5		320	18	5.6		2	2	100.0	687	102	14.8	
≥ 60	158	38	24.1		147	18	12.2		3	0	0	308	56	18.2	
Total	1551	380	24.5	0.20	1161	81	7.0	0.01	24	8	33.3	2736	469	17.1	0.02
Country of birth *(6)*															
Sweden	1211	308	25.4		889	67	7.5		15	2	13.3	2115	377	17.8	
Nordic country (outside Sweden)	65	15	23.1		34	0	0		1	1	100.0	100	16	16.0	
Europe (outside Nordic countries)	168	32	19.0		142	7	4.9		4	2	50.0	314	41	13.1	
Outside Europe	104	25	24.0	0.34	93	7	7.5	-	4	3	75.0	201	35	17.4	0.22
Professional group *(2)*															
Professor	81	32	39.5		203	19	9.4		2	0	0	286	51	17.8	
Senior lecturer	189	63	33.3		193	20	10.4		3	2	67.5	385	85	22.1	
Lecturer and researcher	243	58	23.9		227	11	4.8		5	3	60.0	475	72	15.2	
PhD student	223	41	18.4		170	10	5.9		5	2	40.0	398	53	13.3	
Admin. and technical support staff	758	172	22.7		334	19	5.7		7	1	14.3	1099	192	17.5	
Others	56	14	25.0	< 0.001	33	2	6.1	-	2	0	0	91	16	17.6	0.03
Employment form *(50)*															
Permanent	1111	297	26.7		817	62	7.6		15	4	26.7	1943	363	18.7	
Temporary	420	80	19.0	0.002	314	16	5.1	0.14	9	4	44.4	743	100	13.5	0.001
Managerial position *(18)*															
Yes	175	51	29.1		189	16	8.5		4	1	25.0	360	68	18.5	
No	1369	328	24.0	0.13	961	65	6.8	0.40	20	7	35.0	2350	400	17.0	0.49

^**^P values for chi square regarding difference between subcategories. NB these could not be calculated for the non-binary group due to low number of participants. This was also the case for ‘men and country of birth’ and ‘men and professional group’

^a^*‘Ever experienced’* Experienced at least once during one’s employment at Lund University

As seen in Table 4, presenting corresponding data for the student population, SH was markedly more common in the youngest age groups, i.e., 30 years and younger, both among women (*p* < 0.001) and men (*p* = 0.02). Being born in Sweden was associated with a high prevalence for both genders (28.0% and 11.6%, for women and men, respectively), while for men, SH prevalence was slightly higher among those born in Europe but outside the Nordic countries, 12.4%; however, these differences were statistically significant for women only. Women who were international students reported a prevalence of 22.2% vs. 27.6% among domestic students (*p* = 0.001). The corresponding figures for male students were 9.0% and 11.6% (*p* = 0.12).

**Table 4:** Prevalence of sexual harassment ever experienced^a^, by background factors. Students; *N* = 9667

	Women				Men				Non-binary			All			
	Tot	Yes		p**	Tot			Tot	Yes			Tot			Tot
Background factors *(n of missing)*	n	n	%		n	n	%	*p*	n	n	%	n	n	%	*p*
Age															
18–25	4689	1392	29.7		2752	318	11.6		47	15	31.9	7488	1725	23.0	
26–30	797	180	22.6		472	61	12.9		11	2	18.2	1280	243	19.0	
31–40	347	37	10.7		207	14	6.8		8	2	25.0	562	53	9.4	
≥ 41	222	16	7.2		113	6	5.3		2	1	50.0	337	23	6.8	
Total	6055	1625	26.8	< 0.001	3544	399	11.3	0.02	68	20	29.4	9667	2044	21.1	< 0.001
Country of birth *(6)*															
Sweden	4759	1333	28.0		2854	332	11.6		47	13	27.7	7660	1678	21.9	
Nordic country (outside Sweden)	161	36	22.4		65	4	6.2		3	0	0	229	40	17.5	
Europe (outside Nordic countries)	558	132	23.7		290	36	12.4		12	4	33.3	860	172	20.0	
Outside Europe	574	122	21.3	< 0.001	332	27	8.1	0.13	6	3	50.0	912	152	16.7	< 0.001
International student *(21)*															
Yes	794	176	22.2		402	36	9.0		8	4	50.0	1204	216	17.9	
No	5249	1447	27.6	0.001	3133	363	11.6	0.12	60	16	26.7	8442	1826	21.6	0.003

^**^P values for chi square regarding difference between subcategories. NB these could not be calculated for the non-binary group due to low number of participants

^a^*‘Ever experienced’* Experienced at least once during one’s time as a student at Lund University

In Additional file 3 the frequencies of ever having experienced each of the 10 behaviors are presented in further detail. In general, the same subgroups of staff/PhD students that had affirmed ‘any SH’ to a higher degree (Table 3) reappeared as the most exposed also for each SH behavior. However, a few exceptions could be noted. Stalking was affirmed by as many as 3.2% of women 60 years and older and attempted rape or rape by 1% of women born outside Europe. Men 60 years and older comprised the age group with highest levels of ‘any SH’, but the types of SH deviated from the general pattern among men in that, besides unwelcome comments, unwelcome contact by post or telephone and unwelcome contact online were affirmed by 6.2 and 4.8%, respectively. As regards students, no particular deviations from the general pattern of subgroup exposure described in Table 4 could be noted.

Table 5 presents the distribution of perpetrator gender, stratified by gender of the exposed persons. Among exposed staff/PhD students, 80.4% had been exposed by men, 14.7% by women, none by persons of non-binary gender, and 5.1% by persons of unknown gender. The corresponding figures for students were 82.8%, 16.7%, 0.5%, and 1.9%, respectively. Perpetrators of the same gender as oneself were reported by 2.9% of female versus 17.3% of male staff/PhD students (*p* < 0.001) and by 4.2% of female versus 32.1% of male students (*p* < 0.001). Men were relatively more often than women exposed by persons of unknown gender (*p* < 0.001 for both staff/PhD students and students).

**Table 5:** Gender of the perpetrator/perpetrators^a^

	Gender of participants who had been exposed to SH							
Gender of perpetrators as reported by Staff & PhD students	Women *N* = 380 *(missing* = *8)*		Men *N* = 81 *(missing* = *7)*		Non-binary *N* = 8 *(missing* = *0)*		All *N* = 469 *(missing* = *15)*	
	n	%	n	%	n	%	n	%
Male	356	93.7	14	17.3	7	87.5	377	80.4
Female	11	2.9	57	70.4	1	12.5	69	14.7
Non-binary gender	-	-	-	-	-	-	-	-
Unknown gender	13	3.4	11	13.6	-	-	24	5.1
Gender of perpetrators as reported by Students	Women *N* = 1625 *(missing* = *35)*		Men *N* = 399 *(missing* = *17)*		Non-binary *N* = 20 *(missing* = *0)*		All *N* = 2044 *(missing* = *52)*	
	n	%	n	%	n	%	n	%
Male	1547	95.2	128	32.1	18	90.0	1693	82.8
Female	68	4.2	271	67.9	3	15.0	342	16.7
Non-binary gender	5	0.3	4	1.0	2	10.0	11	0.5
Unknown gender	13	0.8	21	5.3	4	20.0	38	1.9

The percentages are given as percent ‘yes’ out of the total number of exposed persons in each gender group

^a^Exposed persons could mark several options

For staff/PhD students (Table 6), the most commonly reported perpetrator was a university employee, 68.2%. Among exposed women, 43.4% affirmed that this person had a dominant/upper position in relation to themselves. A reverse power relationship was affirmed only by 2.4%. Among male staff/PhD students, the corresponding prevalence regarding the two power situations were 28.4% versus 8.6%; this difference between women and men was statistically significant (*p* = 0.01). In the group of staff/PhD students, as many as 39 (8.3% of all exposed) had experienced a situation with a student as the perpetrator.

**Table 6:** Function and power position of the perpetrator/perpetrators, as reported by Staff & PhD students and students^a^

Perpetrators, as reported by Staff & PhD students		Gender of participants who had been exposed to SH								
		Women *N* = 380		Men *N* = 81		p**	Non-binary *N* = 8		All *N* = 469	
		n	%	n	%		n	%	n	%
University employee		272	71.6	44	54.3		4	50.0	320	68.2
PhD student/research student		34	8.9	12	14.8		1	12.5	47	10.0
Student		26	6.8	11	13.6		2	25.0	39	8.3
Other person that the exposed person met through her/his/their work at the university		80	21.1	13	16.0	0.0013	0	0	93	19.8
Power situation, if the perpetrator was a university employee	The perpetrator had a dominant/upper position	165	43.4	23	28.4		2	25.0	190	40.5
	The perpetrator had a dependent/lower position	9	2.4	7	8.6		1	12.5	17	3.6
	Other person/relationship	118	31.1	17	21.0	0.011	1	12.5	136	29.0
Power situation, if the perpetrator was a student	The perpetrator had a dominant/upper position	4	1.1	0	0		0	0	4	0.9
	The perpetrator had a dependent/lower position	3	0.8	3	3.7		0	0	6	1.3
	Other person/relationship	26	6.8	7	8.6	0.78	1	12.5	34	7.2
Perpetrators, as reported by students		Women *N* = 1625		Men *N* = 399		p**	Non-binary *N* = 20		All *N* = 2044	
		n	%	n	%		n	%	n	%
University employee		104	6.4	17	4.3		1	5.0	122	6.0
PhD student		37	2.3	6	1.5		1	5.0	44	2.2
Person that I met through my work placement		96	5.9	11	2.8		2	10.0	109	5.3
Person, external to Lund University, that I met through my studies		74	4.6	11	2.8		1	5.0	86	4.2
Student		1366	84.1	343	86.0	0.47	19	95.0	1728	84.5
Power situation, if the perpetrator was a university employee	Course director/examiner or Director of studies/Program director	52	3.2	8	2.0		1	5.0	61	3.0
	Other teacher or researcher	51	3.1	5	1.3		1	5.0	57	2.8
	Administrative staff or other employee	20	1.2	6	1.5	0.68	1	5.0	27	1.3
Power situation, if the perpetrator was a student	The perpetrator had a dominant/upper position	266	16.4	43	10.8		3	15.0	312	15.3
	The perpetrator had a dependent/lower position	70	4.3	29	7.3		3	15.0	102	5.0
	Other person/relationship	1172	72.1	288	72.2	0.04	18	90.0	1478	72.3

The percentages are given as percent ‘yes’ out of the total number of exposed persons in each gender group

^**^P values for chi square regarding difference between women and men

^a^Exposed persons could mark several options, and the numbers of missing vary for each question

With regard to students who had affirmed SH (Table 6), the most commonly reported type of perpetrator was another student (84.5%), and in these cases 72% of both women and men reported that there was no particular power relationship involved. Six percent had been exposed to SH by a university employee, 5.3% by a person they encountered through their external placement (e.g. non-university employee, patient, client, etc.), and 4.2% by another external person they met through university activities. Three percent had been exposed to SH by an examiner or other person responsible for the course/program, 2.8% by ‘other teacher or researcher’, and 1.3% by an administrative staff person or other employee. There were no gender differences regarding these results. However, the same gender difference as among staff/PhD students was noted in association with student perpetrators. Women assessed the perpetrator as having a dominant/position more often than the reverse situation, 16.4% vs. 4.3%, while the corresponding figures for men were 10.8% vs. 7.3%, respectively (*p* = 0.04).

All in all, 70.4% of staff/PhD students had experienced SH within the localities of Lund University (Additional file 4), while the corresponding figure for students was 23.5%. Exposure in connection with social activities arranged by student organizations was the most commonly reported setting among students, reported by 72.7%.

## Discussion

The main finding of this study is that experiences of sexual harassment (SH) at the workplace or in connection with studies are common among university employees as well as among students. The pattern of various SH behaviors, as defined by the 10-item instrument used in the current study, was very similar among the two groups. In both groups, exposure to SH was much more common among women and individuals of non-binary gender, compared to men, which is in line with previous findings [3, 35]. Younger age was a predictor among students, but not among staff/PhD students. Particularly among female students, those born in Sweden reported a higher prevalence than those born in another country. In both groups, it was more common that the perpetrator was of opposite gender, although considerably more common among female than among male victims.

The overall prevalence of SH found among staff/PhD students and students in this study was within the expected limits, compared to similar studies [9, 14, 23]. However, whether a single prevalence estimate should be regarded as high or low is a problematic issue, since that depends highly on contextual circumstances. An observed high prevalence could be the result of a high level of occurrence of such behaviors, or alternatively, a greater tendency to regard certain events as violating one’s dignity, or the combination of both. A possible example of the second alternative was discussed in an EU-wide survey (using the same instrument for assessing SH in all participating countries), which revealed that the prevalence of reported SH was considerably higher in EU-countries that score high on the gender equality index, compared to countries characterized by a low score [41]. Thus, in societies with less gender equality, normalization processes regarding SH could lead to underreporting. Processes that influence awareness and reporting should be considered when comparing the prevalence between different contexts as well as comparing changes over time within the same context due to e.g., intervention efforts. In this regard, Sweden is a country with comparatively low gender inequality and less normalization of SH, factors that might have influenced the prevalence currently obtained. Support for this is suggested by the higher SH prevalence shown among Swedish-born female and male students, as well the lower prevalence of SH reported by international students compared to domestic students. Also, the current prevalence is similar to that recently found by Sivertsen among university students in Norway [14], a country also having a low degree of gender inequality.

Apart from the general influence of gender equality in Swedish society, we do not know of any specific ongoing environmental aspects/interventions in the university setting that could have influenced the findings and/or the suggestions we make.

Since sexual orientation of victims and perpetrators was not assessed in this study, we cannot draw any conclusions about whether SH victimization or perpetration is more or less common in any specific sexual orientation group. Regarding sexual identity, the number of individuals who were classified as non-binary was rather low, yet the observed high prevalence indicates their vulnerability for SH as a group.

A difference between staff/PhD students and students was that younger age was associated with a higher reported exposure to SH among students, while the opposite seemed to be the case among staff/PhD students. This could have several explanations. In the general population, younger age is a strong risk factor for SH, which could be more prominently reflected among students, since they are less selected by higher age into their group compared with employees. It could also be the result of a high vulnerability regarding risk for SH among the youngest students due to specific aspects of students’ social life (e.g.,freshman inauguration ceremonies and hierarchical structure of fraternity/nation organizations). Also, we asked for any SH experience ‘ever’ during the participants’ time at the university and this time is probably longer for most staff/PhD than for students, so it might be that staff/PhD were exposed when they were younger.

A notable difference was that staff/PhD students were more often exposed to SH in their daily work environment and within the actual university premises, while the majority of SH events among students occurred outside this context, in the social life of students. This is likely to spur discussions about how far the university’s responsibility regarding prevention and handling of adverse effects of SH among students should be extended. This is important since the victims and perpetrators involved may simultaneously be part of the same work/study environment on the university premises. However, in light of the adverse effects of SH on mental health [3, 4, 42] and the impact of poor mental health on academic performance [43], it is vital that the university and student health services take steps to address SH also when it occurs within the context of students’ social life, rather than solely on university premises. All in all, the results suggest that different intervention strategies may be needed, depending on whether SH takes place inside or outside of the teaching environment.

Our results highlight several important gender-related aspects of SH. First of all, they confirm the well-known observation that when the victim is a woman, the perpetrator most often is a man. The results also showed that when the victim was a man, most commonly it was a woman who was the perpetrator; i.e., the majority of SH situations involved a perpetrator and a victim of opposite gender. However, since SH involving female victims is so much more common than male victims, and since a comparatively higher proportion of perpetrators of male victims are men, the vast majority of perpetrators are men.

The results also revealed which specific types of SH exposure were most prevalent in a university context. It may be noted that according to both staff/PhD students and female students, unwelcome comments, ‘inadvertent’ touching, and suggestive looks/gestures were the most common forms of SH.

We found it somewhat surprising that ‘on-line’ types of SH seemed comparatively uncommon, given the general strong shift in communication from IRL-mode to ICT-mode. Although it is possible that providing more detailed response options could have yielded higher prevalence of “on-line” SH, it is also possible that within the university context. SH primarily takes the form of face-to-face behaviors. This requires further investigation. The information that face-to face behaviors were most frequent is important when discussing the phenomenon of SH in general and for policy and intervention initiatives in particular.

One of the items in the currently used instrument asked about experiences of attempted or completed rape, which are usually defined as sexual violence. This experience was comparatively rare among staff/PhD students (0.4%) and students (1.5%), as opposed to other types of SH. A recent meta-analysis showed that the more frequent and less ‘severe’ types of gender-related workplace hazards were at least as detrimental for women’s well-being as the less frequent but more intense instances of unwanted sexual attention and sexual coercion [35]. It has also been hypothesized that the more conventional and ‘every-day’ forms of SH can be said to constitute the base for normalizing more ‘severe’ types of sexual violence (e.g., attempted or completed rape) [44, 45].

Most SH events among staff/PhD students and students concerned a perpetrator and victim within the same group. When the perpetrator was an employee and a student the victim, the former was usually an employee in a teaching position. Both female and male victims reported that the perpetrator was most often in a position of more power than themselves. However, the opposite situation (the victim was in a superior formal or informal power position) was more commonly reported among men. This pattern was seen among staff/PhD students as well as students. This could perhaps be explained by the theoretical framework proposed by Berdahl, which suggests that SH should be seen as part of a constant negotiation of gender roles and gendered power relations [46]. This negotiation may include both men’s confirmation of traditional male patterns of dominance by condoning already dominant relations as well as revoking women’s conquest of traditionally male dominated positions. However, it may also involve women’s challenging of such traditional patterns by not accepting a subordinate role conveyed through SH behaviors. We do not think that these phenomena are restricted to universities, but they might be more pertinent here, given that a strong feature of this context is the hierarchical formal organization mixed with informal power structures. These structures are often based on career success and network connections, which in the case of SH intersects with the surrounding society’s general gendered power relations, as well as with other types of power relations.

The fact that ‘no particular power relationship’ was reported in most of the cases of students experiencing SH perpetrated by students may be explained by the fact that a large part of SH events took place outside the teaching environment, during student social activities (reported by 73.7% of students, see Additional file 4). Thus, in many cases the perpetrator may well have been unknown to the victim, and consequently, individual power relationships might not have been established.

### Strengths and weaknesses of the study

An important strength of this study is that it is one of very few investigations [9] that assesses both employees and students, who share many of the organizational contextual characteristics of the university environment. This is potentially important for designing appropriate interventions, which to a certain extent are likely to affect both employees and students.

Another strength is that the development of the survey was informed by both focus group discussions and other types of interaction with the target groups. The instrument used for assessing SH captures different concrete situations, which, according to previous research, has been judged to result in more accurate prevalence estimates compared to using only one item [5]. The phrasing of the definition of SH presented to the respondents in the questionnaire reflects well the Swedish legal definition of SH, which we think is valuable when using the results for policy development and for designing interventions in the Swedish context. It was also made very clear to the respondents that the definition of SH refers to *unwelcome* comments and acts.

The main weakness of this study, which it shares with most current surveys, is the low response rate. However, when the general characteristics of the sample (age, gender and type of employment) were checked against register information, the respondents resembled the target population to a very high degree, indicating that the representativity of the sample was reasonable.

A potential limitation is that some PhD students might have been externally financed, i.e., not employed by the university. Due to lack of information, it was not possible to identify them for further analyses. However, since the large majority of PhD students are offered full or part time employment by the university until they have completed their degree, all PhD students were considered to be part of the university staff.

## Conclusions

Exposure to sexual harassment is a fairly prevalent phenomenon in the university environment, both among employees and students. The distribution of SH strongly implies a connection with processes linked to power structures emanating from the wider societal context (gender, age, ethnicity/foreign origin, etc.), as well as those specific to the academic context. Therefore, these power structures should be considered both when designing intervention strategies as well as formulating procedures for case management of reported SH events. In addition, considering the potential adverse impact of SH on academic performance, the results suggest that the university need to address SH even when it occurs within the context of student social life and that different intervention strategies might be needed for students and for employees.

## Supplementary Information

**Additional file 1.**

**Additional file 2.**

**Additional file 3.**

**Additional file 4.**

## Data Availability

Data cannot be shared publicly because of the sensitive nature. Data are available from Lund University (contact via the correspondent author) for researchers who meet the criteria for access to confidential data.
