# Supplementary material for: Sexual harassment among employees and students at a large Swedish university: who are exposed, to what, by whom and where – a cross-sectional prevalence study
Source: BMC Public Health. 2022 Dec 1;22:2240. doi: 10.1186/s12889-022-14502-0 (PMC9714219; doi:10.1186/s12889-022-14502-0)
Supplement: Supplementary file 1 — Additional file 1. [file 12889_2022_14502_MOESM1_ESM.docx]

# Additional File 1

## Assessment of circumstances regarding event/events of sexual harassment (SH)

Since several occasions of SH could have taken place, participants were instructed to select all alternatives that applied when describing the event/events. Therefore the percentages do not add up to 100% in most cases. The percentages are given as percent ‘yes’ out of the total number of exposed persons in each gender group, i.e., among staff/PhD students, out of 380 women, 81 men, and 8 non-binary individuals, and, among students, out of 1625 women, 399 men, and 20 non-binary individuals.

### Perpetrator/perpetrators of SH

The gender of the perpetrator/perpetrators was categorized as ‘male’, ‘female’, ‘non-binary’, and ‘unknown’.

Thereafter, questions were asked about the function of the perpetrator/perpetrators (‘A person employed at Lund University’, ‘A PhD student/research student at Lund University’, ‘A student at Lund University’, or/and ‘Another person who I met through my work at Lund University’) and the relationship between this person and the respondent. Thus, for staff/PhD students, if the perpetrator was an university employee, the following options were offered: a) ‘A person upon whom I was reliant’, b) ‘A person in position of formal seniority to me’, c) ‘A person in a position of power (formal or informal) over me’, d) ‘A person over whom I am/was in a position of (formal or informal) power’, and e) ‘Another person at Lund university’.

The alternatives a), b), and c) were categorized together in the analyses. If the perpetrator was a PhD student, alternatives c), d), and e) were applied.

For students, the following alternatives could be chosen regarding the function of the perpetrator/perpetrators: ‘A Lund University employee’, ‘A PhD student/research student at Lund University’, ‘A person I met through my work placement, (for example internship at a business, hospital, other organization’, ‘A person external to Lund University I met through my studies (external lecturer, researcher from another university, conference participant’, and ‘A student at Lund University’. Further, those having answered ‘A Lund university employee’ were asked whether this person was ‘Course director/examiner for a course in which I participated’, or ‘Director of studies/Program Director’; these two alternatives were categorized together in the analyses. The other response alternatives were ‘Other teacher/researcher’, ‘Administrative staff’, and ‘Other employee’; the two latter were also categorized together in the analyses. Those having answered that the perpetrator was a student were further asked about the relationship, with similar formulations as in alternatives c), d), and e) in the previous paragraph; the first alternative, with a student in a position of power (formal or informal) over oneself, was specified with the wording ‘for example in a higher year, a mentor, a person active in the student life’.

### Location and context

The alternatives given for staff were ‘On Lund University’s premises’, ‘Outside Lund University’s premises in conjunction with an activity connected to my work at Lund University (further education, conference, lunch/dinner/after-work, party, work from home etc.’) and ‘Other ’. For students, the alternatives given were ‘On Lund University’s premises’, ‘Outside Lund University’s premises in conjunction with lectures, internship, exchange, supervision, or equivalent’, and ‘As part of the student social scene (within the union, nation or similar)’.
