# Supplementary material for: Sexual harassment among employees and students at a large Swedish university: who are exposed, to what, by whom and where – a cross-sectional prevalence study
Source: BMC Public Health. 2022 Dec 1;22:2240. doi: 10.1186/s12889-022-14502-0 (PMC9714219; doi:10.1186/s12889-022-14502-0)
Supplement: Supplementary file 2 — Additional file 2. [file 12889_2022_14502_MOESM2_ESM.docx]

# Additional file 2

Prevalence of experiences of sexual harassment (SH)**,* during the last 12 months, 1-3 years ago, and/or more than 3 years ago, as reported by university staff & PhD students and students, in the Tellus survey, Lund University, Sweden, 2020.

|  |  | Exposed last 12 months | | Exposed 1-3 years ago | | Exposed  more than  3 years ago | | Ever exposed** | |
| --- | --- | --- | --- | --- | --- | --- | --- | --- | --- |
| University staff & PhD students | Total Ns | Ns | % | Ns | % | Ns | % | Ns | % |
| Women | 1551 | 119 | 7.7 | 143 | 9.2 | 241 | 15.5 | 380 | 24.5 |
| Men | 1161 | 34 | 2.9 | 29 | 2.5 | 35 | 3.0 | 81 | 7.0 |
| Non-binary | 24 | 3 | 12.5 | 2 | 8.3 | 4 | 16.7 | 8 | 33.3 |
| Total | 2736 | 156 | 5.7 | 174 | 6.4 | 280 | 10.2 | 469 | 17.1 |
| Students |  |  |  |  |  |  |  |  |  |
| Women | 6055 | 1160 | 19.2 | 751 | 12.4 | 239 | 3.9 | 1625 | 26.8 |
| Men | 3544 | 268 | 7.6 | 153 | 4.3 | 57 | 1.6 | 399 | 11.3 |
| Non-binary | 68 | 15 | 22.1 | 7 | 10.3 | 4 | 5.9 | 20 | 29.4 |
| Total | 9667 | 1443 | 14.9 | 911 | 9.4 | 300 | 3.1 | 2044 | 21.1 |

* Exposure=answer ‘yes’ to at least one of the ten questions describing having experienced various sexual harassment behaviors

** Several time frame options could be chosen; ‘ever exposed’ = experienced at least once during one’s employment or time as a student at Lund University
