# Supplementary material for: Sexual harassment among employees and students at a large Swedish university: who are exposed, to what, by whom and where – a cross-sectional prevalence study
Source: BMC Public Health. 2022 Dec 1;22:2240. doi: 10.1186/s12889-022-14502-0 (PMC9714219; doi:10.1186/s12889-022-14502-0)
Supplement: Supplementary file 3 — Additional file 3. [file 12889_2022_14502_MOESM3_ESM.docx]

# Additional file 3 **Type of SH experienced by university staff & PhD students. Women** (N=1551).

Presented as total numbers of participants having experienced each type of SH, and as percentages of each subcategory of participants. Tellus survey, Lund University, Sweden, 2020.

|  |  | Looks, gestures | Pressure for date | ‘Inad-vertent’ | Bodily contact | Gifts | Comments | Post, telephone | Online | Stalking | Rape |
| --- | --- | --- | --- | --- | --- | --- | --- | --- | --- | --- | --- |
| Total ns *(missing)* |  | 231 *(4)* | 106 *(9)* | 131 *(4)* | 80 *(3)* | 38 *(9)* | 256 *(14)* | 52 *(10)* | 60 *(8)* | 28 *(15)* | 6 *(8)* |
|  |  | % | % | % | % | % | % | % | % | % | % |
| Age | Total ns |  |  |  |  |  |  |  |  |  |  |
| ≤ 30 | 188 | 10.7 | 6.9 | 3.7 | 2.1 | 1.6 | 15.4 | 3.2 | 2.7 | 2.1 | 0 |
| 31 – 40 | 373 | 16.7 | 7.3 | 7.0 | 4.0 | 3.0 | 16.8 | 2.7 | 3.2 | 1.4 | 0.3 |
| 41 – 49 | 467 | 16.7 | 6.2 | 10.7 | 5.4 | 2.6 | 18.9 | 3.9 | 4.7 | 1.9 | 0.4 |
| 50 – 59 | 365 | 14.2 | 6.6 | 7.9 | 5.8 | 2.2 | 16.0 | 3.0 | 4.1 | 1.4 | 0.5 |
| ≥ 60 | 158 | 12.2 | 8.3 | 12.3 | 9.5 | 2.6 | 12.7 | 4.4 | 3.8 | 3.2 | 0.6 |
| Total | 1551 | 14.9 | 6.9 | 8.5 | 5.2 | 2.5 | 16.7 | 3.4 | 3.9 | 1.8 | 0.4 |
| Country of birth |  |  |  |  |  |  |  |  |  |  |  |
| Sweden | 1211 | 15.9 | 7.4 | 8.9 | 5.6 | 2.7 | 17.3 | 3.3 | 3.8 | 1.7 | 0.4 |
| Nordic (outside Sweden) | 65 | 9.2 | 7.7 | 3.1 | 1.5 | 3.1 | 14.3 | 6.2 | 6.2 | 4.8 | 0 |
| Europe (outside Nordic countries) | 168 | 10.1 | 3.6 | 4.8 | 4.2 | 1.2 | 13.2 | 2.4 | 2.4 | 0.6 | 0 |
| Outside Europe | 104 | 15.5 | 5.9 | 12.5 | 3.8 | 1.9 | 16.5 | 3.9 | 5.8 | 3.9 | 1.0 |
| Professional group |  |  |  |  |  |  |  |  |  |  |  |
| Professor | 81 | 27.8 | 14.8 | 17.5 | 13.8 | 2.5 | 33.3 | 8.6 | 6.2 | 5.0 | 1.3 |
| Associate professor | 247 | 17.8 | 7.0 | 8.5 | 5.7 | 2.4 | 20.9 | 5.3 | 6.1 | 2.4 | 0.8 |
| Researcher | 185 | 18.4 | 6.5 | 10.3 | 4.9 | 1.1 | 17.4 | 2.7 | 2.7 | 1.1 | 0 |
| PhD student | 223 | 10.4 | 5.9 | 5.8 | 2.7 | 0.9 | 12.1 | 2.3 | 3.6 | 1.3 | 0 |
| Administrative+Techn.staff | 758 | 13.3 | 6.4 | 7.8 | 4.8 | 3.3 | 15.0 | 2.8 | 3.6 | 1.6 | 0.4 |
| Other | 56 | 12.7 | 7.3 | 9.1 | 7.1 | 1.8 | 12.7 | 1.8 | 0 | 1.8 | 0 |
| Employment form |  |  |  |  |  |  |  |  |  |  |  |
| Permanent | 1111 | 16.0 | 7.2 | 9.6 | 6.2 | 2.8 | 18.0 | 3.6 | 4.0 | 1.9 | 0.5 |
| Temporary | 420 | 12.6 | 6.0 | 5.3 | 2.4 | 1.2 | 13.3 | 2.6 | 3.8 | 1.7 | 0 |
| Managerial position |  |  |  |  |  |  |  |  |  |  |  |
| Yes | 175 | 18.4 | 7.6 | 9.1 | 6.9 | 2.3 | 19.8 | 5.1 | 2.9 | 2.3 | 0.6 |
| No | 1369 | 14.5 | 6.7 | 8.4 | 5.0 | 2.5 | 16.3 | 3.2 | 4.0 | 1.8 | 0.4 |

**Type of SH experienced by university staff & PhD students. Men** (N=1161).

Presented as total numbers of participants having experienced each type of SH, and as percentages of each subcategory of participants.

Tellus survey, Lund University, Sweden, 2020.

|  |  | Looks, gestures | Pressure for date | ‘Inad-vertent’ | Bodily contact | Gifts | Comments | Post, telephone | Online | Stalking | Rape |
| --- | --- | --- | --- | --- | --- | --- | --- | --- | --- | --- | --- |
| Total ns *(missing)* |  | 28 *(2)* | 14 *(10)* | 21 *(6)* | 17 *(5)* | 8 *(5)* | 42 *(10)* | 18 *(8)* | 18 *(5)* | 9 *(6)* | 3 *(8)* |
|  |  | % | % | % | % | % | % | % | % | % | % |
| Age | Total ns |  |  |  |  |  |  |  |  |  |  |
| ≤ 30 | 144 | 1.4 | 1.4 | 2.1 | 1.4 | 0 | 1.4 | 0 | 0 | 0 | 0 |
| 31 – 40 | 250 | 1.6 | 0.4 | 2.0 | 1.2 | 0.4 | 2.8 | 1.2 | 0.4 | 0.8 | 0.8 |
| 41 – 49 | 300 | 3.0 | 1.3 | 2.0 | 2.3 | 0.7 | 6.1 | 1.3 | 1.7 | 0.7 | 0.3 |
| 50 – 59 | 320 | 2.8 | 1.3 | 0.9 | 0.3 | 0.9 | 2.8 | 0.6 | 1.6 | 0.9 | 0 |
| ≥ 60 | 147 | 2.7 | 2.1 | 2.7 | 2.7 | 1.4 | 4.1 | 6.2 | 4.8 | 1.4 | 0 |
| Total | 1161 | 2.4 | 1.2 | 1.8 | 1.5 | 0.7 | 3.6 | 1.6 | 1.6 | 0.8 | 0.3 |
| Country of birth |  |  |  |  |  |  |  |  |  |  |  |
| Sweden | 889 | 2.5 | 1.5 | 1.7 | 1.5 | 0.6 | 3.6 | 1.7 | 1.7 | 0.9 | 0.2 |
| Nordic (outside Sweden) | 34 | 0 | 0 | 0 | 0 | 0 | 0 | 0 | 0 | 0 | 0 |
| Europe (outside Nordic countries) | 142 | 2.1 | 0 | 1.4 | 1.4 | 0.7 | 3.6 | 0.7 | 0.7 | 0 | 0 |
| Outside Europe | 93 | 3.2 | 1.1 | 4.3 | 2.2 | 2.2 | 5.4 | 2.2 | 2.2 | 1.1 | 1.1 |
| Professional group |  |  |  |  |  |  |  |  |  |  |  |
| Professor | 203 | 3.4 | 1.0 | 2.0 | 2.0 | 1.0 | 3.5 | 3.5 | 3.5 | 0.5 | 0 |
| Associate professor | 240 | 2.1 | 2.9 | 1.7 | 1.7 | 1.3 | 7.1 | 2.5 | 2.1 | 2.5 | 0.4 |
| Researcher | 180 | 0.6 | 0.6 | 1.1 | 1.1 | 0.6 | 2.8 | 0.6 | 0.6 | 0.6 | 0.6 |
| PhD student | 170 | 1.8 | 1.2 | 3.5 | 1.8 | 0 | 1.8 | 1.2 | 0.6 | 0.6 | 0.6 |
| Administrative+Techn.staff | 334 | 3.0 | 0.6 | 1.2 | 0.9 | 0.6 | 2.4 | 0.6 | 1.2 | 0 | 0 |
| Other | 33 | 6.1 | 0 | 3.1 | 3.0 | 0 | 6.1 | 0 | 0 | 0 | 0 |
| Employment form |  |  |  |  |  |  |  |  |  |  |  |
| Permanent | 817 | 2.8 | 1.4 | 1.8 | 1.6 | 0.9 | 4.2 | 1.7 | 1.6 | 1.0 | 0.2 |
| Temporary | 314 | 1.6 | 0.6 | 1.9 | 1.3 | 0.2 | 2.2 | 1.3 | 0.6 | 0.3 | 0.3 |
| Managerial position |  |  |  |  |  |  |  |  |  |  |  |
| Yes | 189 | 3.2 | 1.6 | 2.1 | 1.6 | 1.1 | 3.7 | 2.7 | 2.7 | 1.6 | 0 |
| No | 961 | 2.3 | 1.2 | 1.8 | 1.5 | 0.6 | 3.7 | 1.4 | 1.4 | 0.6 | 0.3 |

**Type of SH experienced by students. Women** (N=6055).

Presented as total numbers of participants having experienced each type of SH, and as percentages of each subcategory of participants.

Tellus survey, Lund University, Sweden, 2020.

|  |  | Looks, gestures | Pressure for date | ‘Inad-vertent’ | Bodily contact | Gifts | Comments | Post, telephone | Online | Stalking | Rape |
| --- | --- | --- | --- | --- | --- | --- | --- | --- | --- | --- | --- |
| Total ns *(missing)* |  | 1026 *(9)* | 499 *(7)* | 790 *(18)* | 665 *(7)* | 66 *(17)* | 896 *(20)* | 130 *(17)* | 400 *(10)* | 104 *(18)* | 125 *(14)* |
|  |  | % | % | % | % | % | % | % | % | % | % |
| Age | Total ns |  |  |  |  |  |  |  |  |  |  |
| 18 – 25 | 4689 | 18.8 | 9.1 | 14.7 | 12.5 | 1.1 | 16.0 | 2.2 | 7.3 | 1.8 | 2.1 |
| 26 – 30 | 797 | 14.4 | 7.5 | 11.1 | 8.9 | 1.4 | 14.8 | 1.6 | 5.4 | 1.8 | 2.9 |
| 31 – 40 | 347 | 6.1 | 2.9 | 2.3 | 1.2 | 0.6 | 6.4 | 3.2 | 3.2 | 1.2 | 0.3 |
| ≥ 41 | 222 | 4.5 | 2.3 | 2.7 | 2.7 | 0 | 3.6 | 0.5 | 0.9 | 1.4 | 0.5 |
| Total | 6055 | 17.0 | 8.3 | 13.1 | 11.0 | 1.1 | 14.8 | 2.2 | 6.6 | 1.7 | 2.1 |
| Country of birth |  |  |  |  |  |  |  |  |  |  |  |
| Sweden | 4759 | 17.8 | 8.6 | 13.5 | 11.9 | 1.1 | 15.5 | 2.0 | 6.7 | 1.5 | 2.1 |
| Nordic (outside Sweden) | 161 | 13.0 | 9.3 | 13.0 | 9.9 | 1.3 | 10.0 | 1.2 | 5.6 | 4.3 | 4.4 |
| Europe (outside Nordic countries) | 558 | 14.5 | 7.2 | 11.5 | 7.5 | 1.3 | 14.5 | 2.2 | 6.8 | 2.5 | 1.6 |
| Outside Europe | 574 | 13.6 | 5.6 | 10.7 | 7.0 | 0.7 | 11.1 | 3.2 | 5.9 | 1.7 | 1.9 |
| International student |  |  |  |  |  |  |  |  |  |  |  |
| Yes | 794 | 13.4 | 7.7 | 10.6 | 6.3 | 1.1 | 11.7 | 2.3 | 6.7 | 2.3 | 1.9 |
| No | 5249 | 17.6 | 8.3 | 13.5 | 11.7 | 1.1 | 15.3 | 2.1 | 6.6 | 1.6 | 2.1 |

**Type of SH experienced by students. Men** (N=3544).

Presented as total numbers of participants having experienced each type of SH, and as percentages of each subcategory of participants.

Tellus survey, Lund University, Sweden, 2020.

|  |  | Looks, gestures | Pressure for date | ‘Inad-vertent’ | Bodily contact | Gifts | Comments | Post, telephone | Online | Stalking | Rape |
| --- | --- | --- | --- | --- | --- | --- | --- | --- | --- | --- | --- |
| Total ns *(missing)* |  | 167 *(6)* | 71 *(6)* | 167 *(9)* | 191 *(6)* | 12 *(10)* | 136 *(7)* | 23 *(10)* | 55 *(9)* | 23 *(8)* | 20 *(4)* |
|  |  | % | % | % | % | % | % | % | % | % | % |
| Age | Total ns |  |  |  |  |  |  |  |  |  |  |
| 18 – 25 | 2752 | 4.8 | 1.9 | 4.9 | 5.6 | 0.3 | 3.8 | 0.5 | 1.4 | 0.5 | 0.5 |
| 26 – 30 | 472 | 5.1 | 3.2 | 5.5 | 6.4 | 1.1 | 5.1 | 1.5 | 2.5 | 1.5 | 1.1 |
| 31 – 40 | 207 | 2.9 | 1.0 | 2.4 | 1.9 | 0 | 2.9 | 1.5 | 2.4 | 0.5 | 1.0 |
| ≥ 41 | 113 | 5.3 | 1.8 | 1.8 | 1.8 | 0 | 1.8 | 0 | 0 | 0 | 0 |
| Total | 3544 | 4.7 | 2.0 | 4.7 | 5.4 | 0.3 | 3.8 | 0.7 | 1.6 | 0.7 | 0.6 |
| Country of birth |  |  |  |  |  |  |  |  |  |  |  |
| Sweden | 2854 | 4.6 | 2.1 | 4.9 | 6.0 | 0.3 | 3.7 | 0.6 | 1.5 | 0.6 | 0.6 |
| Nordic (outside Sweden) | 65 | 6.2 | 0 | 3.1 | 4.6 | 1.5 | 3.1 | 1.5 | 1.5 | 1.5 | 1.5 |
| Europe (outside Nordic countries) | 290 | 5.5 | 2.4 | 6.6 | 4.5 | 0 | 4.5 | 1.0 | 1.4 | 0 | 0 |
| Outside Europe | 332 | 4.8 | 1.5 | 2.4 | 1.2 | 0.6 | 4.8 | 0.6 | 1.8 | 1.8 | 0.3 |
| International student |  |  |  |  |  |  |  |  |  |  |  |
| Yes | 402 | 4.5 | 2.2 | 3.7 | 2.5 | 0.5 | 4.3 | 1.8 | 2.2 | 0.8 | 0.5 |
| No | 3133 | 4.8 | 2.0 | 4.9 | 5.8 | 0.3 | 3.8 | 0.5 | 1.5 | 0.6 | 0.6 |
