# Supplementary material for: Sexual harassment among employees and students at a large Swedish university: who are exposed, to what, by whom and where – a cross-sectional prevalence study
Source: BMC Public Health. 2022 Dec 1;22:2240. doi: 10.1186/s12889-022-14502-0 (PMC9714219; doi:10.1186/s12889-022-14502-0)
Supplement: Supplementary file 4 — Additional file 4. [file 12889_2022_14502_MOESM4_ESM.docx]

# Additional File 4

Location/context of the exposure/s*, as experienced by university staff & PhD students and by students, who affirmed having been exposed to SH at least once. Tellus survey, Lund University, Sweden, 2020.

| Staff & PhD students | Participants who had been exposed to SH | | | | | | | | | | | | |
| --- | --- | --- | --- | --- | --- | --- | --- | --- | --- | --- | --- | --- | --- |
|  | Women  N=380  *(missing=10)* | | Men  N=81  *(missing=9)* | | | Non-binary  N=8  *(missing=0)* | | | | All  N=469  *(missing=19)* | | | |
|  | ns | % | ns | % | | ns | | % | | ns | | % | |
| On Lund University’s premises | 270 | 71.1 | 53 | 65.4 | | 7 | | 87.5 | | 330 | | 70.4 | |
| Outside Lund University’s premises in conjunction with an activity connected to my work at Lund University | 133 | 35.0 | 17 | 21.0 | | 0 | | 0 | | 150 | | 32.0 | |
| Other location | 31 | 8.2 | 11 | 13.6 | | 3 | | 37.5 | | 45 | | 9.6 | |
|  | | | | | | | | | | | | | |
| Students | Women  N=1625  *(missing=49)* | | Men  N=399  *(missing=22)* | | | | Non-binary  N=20  *(missing=0)* | | | | All  N=2044  *(missing=71)* | | |
|  | ns | % | ns | | % | | ns | | % | | ns | | % |
| On Lund University’s premises | 389 | 23.9 | 85 | | 21.3 | | 6 | | 30.0 | | 480 | | 23.5 |
| Outside Lund University’s premises in conjunction with lectures, internship, exchange, supervision, or equivalent | 192 | 11.8 | 32 | | 8.0 | | 3 | | 15.0 | | 227 | | 11.1 |
| As part of the student social scene | 1188 | 73.1 | 285 | | 71.4 | | 14 | | 70.0 | | 1487 | | 72.7 |

* Exposed persons could mark several options.

The percentages are given as percent ‘yes’ out of the total number of exposed persons in each gender group.
